# Does chronic disease influence susceptibility to the effects of air pollution on depressive symptoms in China?

**DOI:** 10.1186/s13033-018-0212-4

**Published:** 2018-06-18

**Authors:** Qing Wang, Zhiming Yang

**Affiliations:** 1 School of Business, Dalian University of Technology, Panjin, 124221 Liaoning China; 2 Donlinks School of Economics and Management, University of Science and Technology Beijing, Beijing, 100083 China

**Keywords:** Air pollution, Depression, Chronic diseases, China

## Abstract

**Background:**

Exogenous stressors resulting from air pollution can lead to depression and chronic disease. Chinese levels of air pollution are among the highest in the world, and although associated adverse health effects are gradually emerging, research determining individual vulnerability is limited. This study estimated the association between air pollution and depressive symptoms and identified whether chronic disease influences an individual’s susceptibility to depressive symptoms relating to air pollution.

**Methods:**

Individual sample data from the China Health and Retirement Longitudinal Study and a group of city-level variables in 2011 and 2013 were used with the random effects model and Tobit model. Adjustments were made for demographic, socioeconomic status, health behavior, and city-level climate variables with respect to living areas. Analysis was also stratified using chronic disease characteristics.

**Results:**

The total Center for Epidemiological Studies Depression scale evaluating depressive symptoms ranged between 7 and 28 [average 11.623 (SD = 4.664)]. An 1% increase in sulfur dioxide and total suspended particulate emission intensities was associated with depressive symptoms scores that were 1.266 (SE = 0.107, P < 0.001, 95% CI 1.057–1.475) and 1.318 (SE = 0.082, P < 0.001, 95% CI 1.157–1.480) higher, respectively. Compared to respondents without chronic disease, those with chronic diseases such as hypertension, dyslipidemia, diabetes or high blood sugar, cardiovascular diseases, cancer or malignant tumor, liver disease, chronic lung diseases, kidney disease, stomach or other digestive disease, arthritis or rheumatism, and asthma had scores that were higher for depressive symptoms.

**Conclusions:**

Results confirm that the adverse health effects of air pollution should be considered when developing air pollution policies. Findings also provide justification for mental health interventions targeting air pollution exposure, especially for people with chronic diseases.

## Background

The Chinese government encouraged the growth of industries and urbanization since 1978. However, the associated rapid economic development has caused environmental issues, and China is now one of the most polluted countries in the world [1–3]. For example, the annual average Total Suspended Particulates (TSP) concentration regularly exceeds 400 μg/m^3^ in China [4], which is significantly higher than that in large European cities (e.g. Oslo, 15 μg/m^3^; Marseille, 18 μg/m^3^) [5] and World Health Organization (WHO) primary (80 μg/m^3^) and secondary (60 μg/m^3^) standards [6, 7].

Air pollution is believed to be associated with depressive symptoms [8]. Potential biological mechanisms relating to depressive symptoms include reactivity to exogenous stressors; alterations of neurohumoral, immune, and autonomic regulation; dysfunction of neuro transmitter systems; and oxidative stress [9]. Cell cultures and experimental animals studies have shown neuropathological effects from air pollution exposure [10–12], and previous empirical studies have observed that air pollution increases the prevalence of depressive symptoms in Korea, Japan, and the Netherlands [8, 13–15]. Furthermore, an increasing number of emergency department visits for depression in Canada and Korea have been documented [16, 17].

Depression is a serious problem in China [18, 19 ]: in 2013, 36 million years of healthy life were lost to mental illness in China, and estimates suggest that by 2025, 39.6 million years of healthy life will be lost (10% increase) [20]. Although little is known about the association between air pollution and depression, several Chinese studies have found a relationship between exposure to air pollution and happiness [21, 22], depressive symptoms [23], cognitive functions [24, 25] and hospital admissions for mental disorders [26], when results were adjusted for demographics and socioeconomic status. It is considered that a further decline in air quality could cause an increased risk to health and an associated increase in depressive symptoms. Therefore, this study uses nationally representative data to estimate the association between air pollution and depression measured by the Center for Epidemiologic Studies Depression (CES-D) scale.

Air pollution regulations based on observed health effects in the general population may be insufficient to protect exceptionally vulnerable subgroups. Inconsistent study results have been found within past study cohorts. For example, no significant association between air pollution exposure and depressive symptoms was found in a Boston-area study [27], although other American studies reported that exposure to air pollution was related to anxiety symptoms [28], which often have a comorbidity with depression [29]. One American study found that stroke victims were more susceptible to the effects of air pollution with respect to cognitive functions [30]. Thus, it is believed that chronic disease (e.g. hypertension), which is often regarded as a marker of inflammation and vascular dysfunction, may mediate an association between air pollution and depressive symptoms [31, 32]. Compared to people in good physical health, the well-known adverse mental health effects of air pollution may mean that respondents with chronic disease are likely to believe that their physical health is being damaged [33–35]. However, the role of an individual’s physical health status in the association between air pollution and depression symptoms has not yet been addressed in China. Therefore, this study aims to assess which individuals have a greater vulnerability to the adverse effects of air pollution [21].

## Data and methods

### Data

Individual sample data and a group of city-level variables were obtained to evaluate the relationship between air pollution, chronic disease, and depressive symptoms. Individual data were collected from CHARLS 2011 and 2013, which were national representative surveys conducted with middle-aged and elderly Chinese residents (aged 45 years and above) using face-to-face computer-assisted personal interviews. The CHARLS questionnaire included the following modules: demographics, family structure/transfer, health status and functioning, biomarkers, health care and insurance, work, retirement and pension, income and consumption, assets (individual and household), and community-level information. These surveys were approved by the ethics committee of the Institutional Review Board of Peking University.

Using multi-stage stratified probability-proportionate-to-size sampling, the sample in CHARLS represented approximately 10,000 households in 150 counties/districts (a total of 450 villages/resident communities). The baseline survey was conducted between June 2011 and March 2012 with a response rate of 80.5% and a total sample of 17,545 respondents. A total of 15,020 (86%) respondents participated in the follow-up survey in 2013, but 2525 (14%) respondents had died or declined participation in the study. In this study, CHARLS 2011 and 2013 panel data were constructed to estimate the relationship between air pollution and depressive symptoms for 15,020 respondents (15,020 × 2 = 30,040 samples). Of the respondents, 47% were male with a mean age of 60 years. Ages and gender distribution were very similar to those in the 6th national census conducted in 2010 [36].

City-level variables included monthly temperature, monthly relative humidity, and annual air pollution. Daily meteorological data from 839 stations in 2011 and 2013 were collected from the China Meteorological Science Data Sharing Service Network—China Ground Climate Daily Data. The station-level data were aggregated at a city level by matching stations to the closest city based on the exact longitude and latitude of the weather station and the longitude and latitude of the county centroid. The average monthly temperature and relative humidity of CHARLS 125 sample cities were then calculated from daily data. Based on survey city and month, the results from 15,020 respondents in the two CHARLS waves were combined with the meteorological data from 125 cities.

**Fig. 1:**
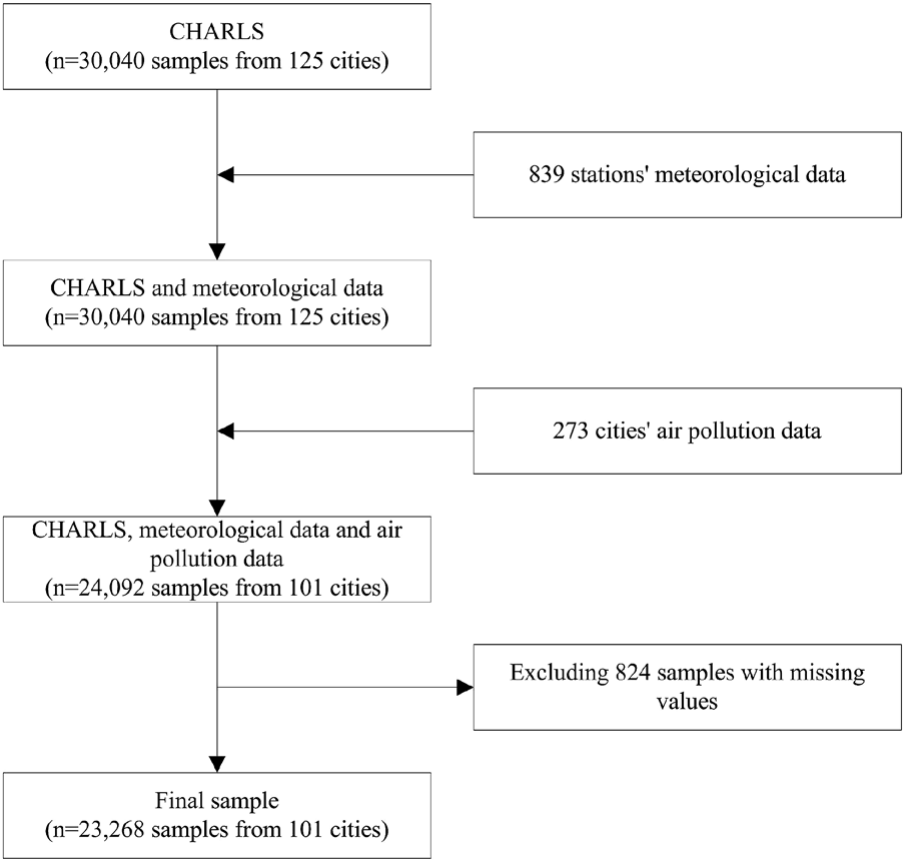
Flow chart

The annual sulfur dioxide (SO_2_) and TSP emissions from 273 cities in 2011 and 2013 were obtained from the 2012 and 2014 China City Statistical Yearbook. Based on survey year and city, 12,046 respondents of the 15,020 respondents in the two CHARLS waves were matched to the air pollution data from 101 cities. After excluding 412 respondents that provided missing values from 12,046 respondents, 11,634 respondents were included, and the final sample size was 23,268 (11,634 × 2) samples from 101 cities. Figure 1 presents a flow chart of the study process.

### Variables

#### Depressive symptoms

A modified seven-item version of the CES-D scale was constructed to measure depressive symptoms [37]. Respondents reported the frequency of experiencing the following seven depressive symptoms during the past week: (1) “was bothered by things,” (2) “had trouble keeping mind on what was doing,” (3) “felt depressed,” (4) “felt everything he/she did was an effort,” (5) “felt fearful,” (6) “sleep was restless,” and (7) “felt lonely.” Each answer was encoded from 1 to 4: 1 = rarely or none, 2 = some or a little, 3 = occasionally or a moderate amount, and 4 = most or all of the time, with total scores ranging from 7 to 28. A summed score of the seven items was calculated, with lower scores indicating fewer depressive symptoms. This shortened seven-item CES-D scale is a widely used indicator for depressive symptoms [38, 39]. Its validity, reliability, and cultural equivalence have been proven in China [40]. In our data set, CES-D was also demonstrated to have high internal consistency (Cronbach’s alpha = 0.82) and to construct validity (Kaiser–Meyer–Olkin = 0.87) according to the standards of Meulen et al. [41], Kara [42], and Aly [43], which suggest that if the Cronbach’s alpha and Kaiser–Meyer–Olkin test value exceeds the recommended level of 0.70 then data is considered to be highly reliable [41–43].

#### Air pollution

Following previous studies, SO_2_ and TSP emission intensity, SO_2_ and TSP emissions per capita, and SO_2_ and TSP emissions per unit area were calculated to measure air pollution [44–47]. Pollution intensity refers to the indicator of pollution emissions per industrial gross domestic product (GDP) (industrial economic output). Log transformation of air pollution data was applied to minimize skewness [45].

#### Chronic disease

A categorical variable for a doctor to use in diagnosing chronic disease was created based on the question, “Have you ever been diagnosed with hypertension, dyslipidemia, diabetes or high blood sugar, cardiovascular disease (heart attack, coronary heart disease, angina, congestive heart failure, stroke or other heart problems), cancer or malignant tumor, liver disease, chronic lung disease, kidney disease, stomach or other digestive disease, arthritis or rheumatism and asthma by a doctor?” The variable equaled 1 or 0 for respective replied of “yes” or “no”.

### Estimation Strategy

Descriptive analysis was first conducted to describe sample characteristics of the total sample and for chronic disease status. Frequencies with percentages were presented for categorical variables (gender, marital status, education, employment status, insurance status) and means with standard deviations for continuous variables (CES-D, air pollution indicators, climate indices, income, and age). P-values were calculated using the Chi square test for categorical variables, and one-way Analysis of Variance (ANOVA) for continuous variables between groups (with or without chronic disease).

A random effects model was then applied to link air pollution intensity with depressive symptoms. Omitted variable bias was controlled using the random effects model. The individual random effects model is presented as follows,

1 $$Depressive_{ijt} = a_{0} + Air_{jt} a_{ 1} + X_{ijt} a_{ 2} + u_{i} + v_{t} + e_{ijt} ,$$

where *a*_0_, *a*_1_, *a*_2_ are the parameters to be estimated; *u*_*i*_ and *v*_*t*_ are the individual effects and year fixed effects, respectively; *e*_*ijt*_ is the idiosyncratic error term; and *Depressive*_*ijt*_ is the depressive symptoms of person *i* in city *j* in year *t*; *Air*_*jt*_ is a variable indicating the log of air pollution intensity, *X*_*ijt*_ represents an individual’s demographic, socioeconomic status, health behavior, and city-level climate variables in the living area. To be more specific, the demographic variables included whether male or female, marital status [reference group: married with spouse present (common-law marriage was considered married)], and age; socioeconomic status was measured by household income per capita, education level, employment status, insurance status, and rural/urban residence; educational attainment was defined at four levels (informal education, informal education but can read and write, primary school, and junior high school and above), and a categorical variable for educational attainment with four values was constructed, with informal education serving as the reference group; household income was divided by the number of household members and household income per capita was subsequently ranked and divided into five pentiles, with the lowest group serving as a reference; employment status was categorized into three groups: unemployed (including retired), self-employed, and wage earner; for health insurance coverage, respondents were recoded into a dummy variable with three values [the urban employee-based basic medical insurance scheme (UEBMI), the rural new cooperative medical scheme (NCMS), and the urban resident-based basic medical insurance scheme (URBMI)], with uninsured respondents as the reference group; current smoker and drinker were included as indicators of the respondents’ current health behaviors; and city-level average monthly temperature, relative humidity, and city dummy variables were also controlled.

The interaction between chronic disease and air pollution intensity was then controlled in multivariate regression to establish whether chronic disease influences an individual’s susceptibility to depressive symptoms with respect to air pollution,

2 $$Depressive_{ijt} = b_{0} + Air_{jt} b_{ 1} + Chronic_{ijt} b_{ 2} + Air_{jt} \times Chronic_{ijt} b_{ 3} + X_{ijt} b_{ 4} + u_{i} + v_{t} + e_{ijt} ,$$

where *b*_0_, *b*_1_, *b*_2_, *b*_3_, *b*_4_ are the parameters to be estimated; *Chronic*_*ijt*_ is a dummy variable indicating whether a respondent has a chronic disease; and *Air*_*jt*_× *Chronic*_*ijt*_ is the interaction between chronic disease and air pollution intensity after decentralization. Decentralization of air pollution indicators was calculated by subtracting the mean of city-level air pollution intensity from air pollution intensity in each city and then dividing it by the standard deviation of city-level air pollution intensity using the center-command in Stata 14 [48].

Analyses were then stratified according to chronic disease characteristics. Depressive symptoms may also affect an individual’s physical health status and lead to endogenous issues [49]. Stratification was conducted to eliminate any possible endogenous issues by excluding respondents with chronic diseases (hypertension, dyslipidemia, diabetes or high blood sugar, cardiovascular diseases, arthritis and asthma) because such diseases may stem from depressive symptoms. Under these conditions no other possible methods were available for now.

The total CES-D scale used to evaluate depressive symptoms of middle-aged and elderly individuals ranged between 7 and 28, which represents a limited dependent variable. Therefore, least square regression was directly applied to render inconsistent estimates [50, 51]. The Tobit model was used for robust analysis in this respect, and marginal effects were reported,

3 $$Depressive^{*}_{ijt} = c_{0} + Air_{jt} c_{ 1} + Chronic_{ijt} c_{ 2} + Air_{jt} \times Chronic_{ijt} c_{ 3} + X_{ijt} c_{ 4} + u_{i} + v_{t} + e_{ijt} ,$$

4 $$Depressive_{ijt} = { 7}\quad if \, Depressive^{*}_{ijt} \le 7,$$

5 $$Depressive_{ijt} = Depressive^{*}_{ijt} \quad if {\,} 7< Depressive^{*}_{ijt} < 2 8,$$

6 $$Depressive_{ijt} = { 28}\quad if {\,} Depressive^{*}_{ijt} \ge 2 8,$$

where *c*_0_, *c*_1_, *c*_2_, *c*_3_, *c*_4_ are the parameters to be estimated; $Depressive_{ijt}^{*}$ is a latent variable and *Depressive*_*ijt*_ is its observable variable. A robust standard error was derived using bootstrapping methods and conducting 500 bootstrap replications.

In addition, by using the Tobit model with stratified samples, the effects of SO_2_ and TSP emission per capita/per unit area on depression were regressed (and are presented in Appendix: Table 4). The results were consistent with those using air pollution emission intensity. Stata version 14 was used for all analyses [48].

## Results

Participant characteristics and average air pollutant intensity across chronic disease are shown in Table 1. On average, SO_2_ and TSP emissions accounted for 82.950 (SD = 78.355) and 45.571 (SD = 49.025) tons per 100 million Chinese yuan of industrial GDP, respectively. SO_2_ emissions per unit area and per capita were 6.812 (SD = 8.307) tons/km^2^ and 135.137 (SD = 120.211) tons/10,000 people; and TSP emissions per unit area and per capita were 3.079 (SD = 3.495) tons/km^2^ and 70.938 (SD = 81.064) tons/10,000 people; and average temperature and humidity were 26.599 °C (SD = 3.935) and 73.253% (SD = 7.603), respectively.

**Table 1:** Statistical description

Variable	All sample (N = 23,268)		Group with chronic disease (N = 15,412)		Group without chronic disease (N = 7856)		Group with chronic disease VS Group without chronic disease
	Mean	Std. Dev.	Mean	Std. Dev.	Mean	Std. Dev.	P-value^a^
Air pollution emission intensity (tons per 100 million Chinese yuan)							
SO_2_	82.950	78.355	82.602	74.980	83.632	84.587	< 0.001
TSP	45.571	49.025	46.510	50.816	43.729	45.253	< 0.001
Air pollution emission per unit area (tons/km^2^)							
SO_2_	6.812	8.307	6.667	8.728	7.097	7.403	< 0.001
TSP	3.079	3.495	2.998	3.576	3.236	3.326	< 0.001
Air pollution emission per capita (tons/10,000 people)							
SO_2_	135.137	120.211	132.127	118.256	141.042	123.753	< 0.001
TSP	70.938	81.064	70.173	80.964	72.440	81.243	< 0.001
Climatic indexes							
Average monthly temperature (0.1 °C)	265.990	39.353	266.038	39.678	265.894	38.710	< 0.001
Average monthly relative humidity (%)	73.253	7.603	73.077	7.634	73.599	7.529	< 0.001
Depressive symptoms	11.623	4.664	12.176	4.858	10.538	4.043	< 0.001
Age	60.101	9.989	61.101	9.834	58.139	10.000	< 0.001
Income (Chinese yuan/year)	8175	14,912	7970	13,343	8576	17,583	0.011

	n	%	n	%	n	%	P-value^b^
Male	11,108	47.739	7165	46.490	3943	50.191	0.010
Unmarried	2854	12.266	1991	12.919	863	10.985	0.022
Living in urban area	9016	38.748	5947	38.587	3069	39.066	0.730
Education							< 0.001
No education	5973	25.670	4145	26.895	1828	23.269	
No education but can read/write	4218	18.128	2946	19.115	1272	16.191	
Primary school	5181	22.267	3498	22.696	1683	21.423	
Junior high school and above	7896	33.935	4823	31.294	3073	39.117	
Employment status							< 0.001
Unemployed	7734	33.239	5623	36.485	2111	26.871	
Self-employed	10,162	43.674	6710	43.537	3452	43.941	
Wage earner	5372	23.087	3079	19.978	2293	29.188	
Insurance							0.037
Uninsured	1455	6.253	854	5.541	601	7.650	
NCMS and URBMI	18,635	80.089	12,389	80.385	6246	79.506	
UEBMI	3178	13.658	2169	14.074	1009	12.844	
Health behavior							
Current Smoker	7713	33.149	4757	30.866	2956	37.627	< 0.001
Current Drinker	7756	33.333	4932	32.001	2824	35.947	0.005

^a^Frequencies with percentages were presented for categorical variables. P-values were calculated by Chi square test between groups having chronic disease or not

^b^Means with standard deviations were presented for continuous variables, and P-values were calculated one-way ANOVA between groups having chronic disease or not

The mean age of respondents was 60 (SD = 9.989) years; 48% of respondents (11,108/23,268) were male; 88% (20,414/23,268) were married or cohabiting; 39% (9016/23,268) lived in an urban area; 44% (10,191/23,268) of respondents had no formal education; 33% (7734/23,268) were unemployed; 44% (10,162/23,268) were self-employed; and the majority had health insurance [94% (21,813/23,268)]. A total of 33% (7713/23,268) reported smoking and 33% (7756/23,268) reported drinking alcohol. The respondents earned an average of 8175 (SD = 14,912) Chinese yuan per year per capita. The average depressive symptoms score was 11.623 (SD = 4.664). Compared to participants with chronic disease, those without chronic disease were more likely to report lower depressive symptoms [12.176 (SD = 4.858) versus 10.538 (SD = 4.043)] but not more likely to be exposed to air pollution.

Models 1 and 3 from Table 2 show the correlation between air pollution and depressive symptoms in China after adjusting for multiple covariates. Increasing levels of air pollution were found to be significantly associated with higher depressive symptoms: an increase in SO_2_ and TSP emission intensities of 1% was associated with increasing depressive symptoms scores by 1.266 (SE = 0.107, P < 0.001, 95% CI 1.057–1.475) and 1.318 (SE = 0.082, P < 0.001, 95% CI 1.157–1.480), respectively. Models 2 and 4 from Table 2 present the interaction between air pollution and chronic disease and their effect on depressive symptoms. After controlling for the interaction of air pollution and chronic disease, a positive correlation between air pollution and depressive symptoms was observed, as expected. An 1% increase in the intensities of SO_2_ and TSP emissions was associated with 1.237 (1.093 (SE = 0.116, P < 0.001, 95% CI 0.866–1.320) + 0.217 (SE = 0.084, P = 0.009, 95% CI 0.053–0.380) × 15,412/23,268 = 1.093 + 0.217 × 66%) and 1.301 (1.115 (SE = 0.092, P < 0.001, 95% CI 0.934–1.296) + 0.281 (SE = 0.071, P < 0.001, 95% CI 0.143–0.420) × 66%) higher depressive symptoms scores, respectively. In addition, due to an 1% increase in the intensities of SO_2_ and TSP emissions, the depressive symptoms scores for respondents with chronic disease increased by 1.903 (1.384 (SE = 0.068, P < 0.001, 95% CI 1.250–1.518) + 0.217 (SE = 0.084, P = 0.009, 95% CI 0.053–0.380) × log(82.950)) and 1.854 (1.388 (SE = 0.068, P < 0.001, 95% CI 1.254–1.522) + 0.281 (SE = 0.071, P < 0.001, 95% CI 0.143–0.420) × log(45.571)), respectively. Given the same intensities of SO_2_ and TSP emissions, respondents with chronic disease had higher scores for depressive symptoms by 0.217 (SE = 0.084, P = 0.009, 95% CI 0.053–0.380) and 0.281 (SE = 0.071, P < 0.001, 95% CI 0.143–0.420) than those without chronic disease.

**Table 2:** Association of air pollution intensity and depressive symptoms and the role of chronic disease (N = 23,268)

Variables	Influence of SO_2_ emission intensity on depressive symptoms				Influence of TSP emission intensity on depressive symptoms			
	Model 1		Model 2		Model 3		Model 4	
	Coef.	Std. Err.	Coef.	Std. Err.	Coef.	Std. Err.	Coef.	Std. Err.
Log of SO_2_ intensity	1.266***	0.107	1.093***	0.116	–	–	–	–
Log of TSP intensity	–	–	–	–	1.318***	0.082	1.115***	0.092
Chronic disease	–	–	1.384***	0.068	–	–	1.388***	0.068
Log of SO_2_ intensity × Chronic disease	–	–	0.217***	0.084	–	–	–	–
Log of TSP intensity × Chronic disease	–	–	–	–	–	–	0.281***	0.071
Log of average monthly temperature	0.189**	0.085	0.176**	0.085	0.201**	0.087	0.191**	0.087
Log of average monthly relative humidity	− 0.690**	0.333	− 0.693**	0.332	− 0.445	0.335	− 0.451	0.333
Age group								
50–59	0.248***	0.090	0.134	0.089	0.263***	0.090	0.148*	0.089
60–69	0.148	0.105	− 0.057	0.104	0.174*	0.104	− 0.031	0.103
More than 70	− 0.396***	0.131	− 0.606***	0.130	− 0.368***	0.131	− 0.578***	0.130
Male	1.270***	0.091	1.269***	0.090	1.282***	0.091	1.283***	0.090
Unmarried	0.680***	0.121	0.701***	0.119	0.682***	0.121	0.701***	0.119
Living in urban area	− 0.400***	0.104	− 0.398***	0.103	− 0.395***	0.104	− 0.387***	0.103
Education								
No education but can read/write	0.414***	0.118	0.373***	0.116	0.416***	0.118	0.376***	0.116
Primary school	− 0.043	0.112	− 0.081	0.110	− 0.039	0.112	− 0.077	0.110
Junior high school and above	− 0.470***	0.114	− 0.454***	0.112	− 0.461***	0.114	− 0.448***	0.112
Employment status								
Self− employed	− 0.165*	0.089	− 0.109	0.088	− 0.152*	0.088	− 0.096	0.088
Wage earner	− 0.351***	0.090	− 0.262***	0.089	− 0.354***	0.090	− 0.265***	0.089
Insurance								
NCMS and URBMI	− 0.061	0.130	− 0.123	0.129	− 0.052	0.129	− 0.113	0.128
UEMBI	− 0.432***	0.152	− 0.520***	0.151	− 0.418***	0.151	− 0.503***	0.150
Income group								
21–40th percentile	0.328***	0.100	0.335***	0.099	0.340***	0.100	0.349***	0.099
41–60th percentile	0.078	0.096	0.068	0.095	0.091	0.096	0.082	0.095
61–80th percentile	− 0.073	0.096	− 0.068	0.095	− 0.063	0.096	− 0.056	0.095
81–100th percentile	− 0.289***	0.101	− 0.296***	0.100	− 0.288***	0.101	− 0.295***	0.100
Health behavior								
Current drinker	0.040	0.072	0.108	0.072	0.0459	0.072	0.114	0.072
Current smoker	− 0.196**	0.081	− 0.156*	0.080	− 0.186**	0.081	− 0.144*	0.080
Constant	7.165***	1.548	7.025***	1.562	7.219***	1.538	7.079***	1.544
City dummy variables	YES		YES		YES		YES	
Adjusted R^2^	0.139		0.165		0.139		0.165	
Wald Chi square	2126***		2596***		2228***		2707***	

Models 1–4 are estimated using the xi:xtreg-command in Stata 14. Decentralization was calculated using the center-command

* P < 0.10; ** P < 0.05; *** P < 0.01

Models 1–4 from Table 3 show the results stratified using chronic disease characteristics; models 5–8 show results using the Tobit model; models 9–12 show the results stratified using chronic disease characteristics and the Tobit model. Robust analysis shows that the results obtained were consistent with those using the random effects model. According to models 10 and 12 from Table 3, for individuals with cancer or malignant tumor, chronic lung diseases, liver diseases, kidney disease, and stomach diseases, when the SO_2_ and TSP emission intensities increased by 1% individuals showed an increase in depressive symptom scores of 0.844 (0.788 (SE = 0.128, P < 0.001, 95% CI 0.537–1.039) + 0.221 (SE = 0.091, P = 0.015, 95% CI 0.044–0.399) × 2669/10,508 = (0.788 + 0.221 × 25%) and 0.818 (0.765 (SE = 0.118, P < 0.001, 95% CI 0.534–0.997) + 0.208 (SE = 0.107, P = 0.051, 95% CI 0–0.417) × 25%), respectively. In addition, due to an 1% increase in the intensities of SO_2_ and TSP emissions, respondents with these chronic diseases scored higher for depressive symptoms by 1.292 (0.869 (SE = 0.102, P < 0.001, 95% CI 0.668–1.069) + 0.221 (SE = 0.091, P = 0.015, 95% CI 0.044–0.399) × log (82.000)) and 1.208 (0.866 (SE = 0.114, P < 0.001, 95% CI 0.643–1.090) + 0.208 (SE = 0.107, P = 0.051, 95% CI 0–0.417) × log(43.885)), respectively. Given the same intensities of SO_2_ and TSP emissions, individuals with chronic disease had higher scores for depressive symptoms by 0.221 (SE = 0.091, P = 0.015, 95% CI 0.044–0.399) and 0.208 (SE = 0.107, P = 0.051, 95% CI 0–0.417) than those without chronic disease. However, the impacts of air pollution were reduced after eliminating the endogenous variable, which supports the hypothesis that depressive symptoms influence physical health.

**Table 3:** Association of air pollution intensity and depressive symptoms and the role of chronic disease: Robustness test

Variables	Influence of SO_2_ emission intensity on depressive symptoms				Influence of TSP emission intensity on depressive symptoms			
	Model 1		Model 2		Model 3		Model 4	
	Coef.	Std. Err.	Coef.	Std. Err.	Coef.	Std. Err.	Coef.	Std. Err.
Association of air pollution intensity and depressive symptoms and the role of chronic disease among respondents without mental-related chronic disease (N = 10,508)								
Log of SO_2_ intensity	0.952***	0.141	0.901***	0.145	–	–	–	–
Log of TSP intensity	–	–	–	–	0.929***	0.115	0.875***	0.118
Chronic disease	–	–	0.993***	0.113	–	–	0.991***	0.113
Log of SO_2_ intensity × chronic disease	–	–	0.256*	0.138	–	–	–	–
Log of TSP intensity × chronic disease	–	–	–	–	–	–	0.240**	0.118
Adjusted R^2^	0.123		0.139		0.123		0.140	
Wald Chi square	862***		961***		876***		974***	

	Model 5		Model 6		Model 7		Model 8	
	Marginal effects	Boot. Std.	Marginal effects	Boot. Std.	Marginal effects	Boot. Std.	Marginal effects	Boot. Std.
Association of air pollution intensity and depressive symptoms and the role of chronic disease: Tobit model (N = 23,268)								
Log of SO_2_ intensity	1.122***	0.101	0.969***	0.090	–	–	–	–
Log of TSP intensity	–	–	–	–	1.169***	0.071	0.989***	0.068
Chronic disease	–	–	1.227***	0.056	–	–	1.230***	0.065
Log of SO_2_ intensity × chronic disease	–	–	0.191***	0.063	–	–	–	–
Log of TSP intensity × chronic disease	–	–	–	–	–	–	0.248***	0.061
Sigma(u)	2.484***	0.054	2.406***	0.038	2.491***	0.042	2.414***	0.040
Sigma(e)	3.663***	0.029	3.661***	0.024	3.653***	0.037	3.650***	0.030
Log likelihood	− 67,019		− 66,835		− 66,985		− 66,798	

	Model 9		Model 10		Model 11		Model 12	
	Marginal effects	Boot. Std.	Marginal effects	Boot. Std.	Marginal effects	Boot. Std.	Marginal effects	Boot. Std.
Association of air pollution intensity and depressive symptoms and the role of chronic disease among respondents without mental-related chronic disease: Tobit model (N = 10,508)								
Log of SO_2_ intensity	0.832***	0.122	0.788***	0.128	–	–	–	–
Log of TSP intensity	–	–	–	–	0.812***	0.102	0.765***	0.118
Chronic disease	–	–	0.869***	0.102	–	–	0.866***	0.114
Log of SO_2_ intensity × chronic disease	–	–	0.221**	0.091	–	–	–	–
Log of TSP intensity × chronic disease	–	–	–	–	–	–	0.208**	0.107
Sigma(u)	2.098***	0.068	2.052***	0.064	2.103***	0.071	2.056***	0.070
Sigma(e)	3.440***	0.042	3.441***	0.050	3.434***	0.038	3.435***	0.048
Log likelihood	− 29,353		− 29,307		− 29,344		− 29,297	

Models 1–4 are estimated using the xi:xtreg-command in Stata 14, models 5–12 are estimated using the xi:xttobit-command. Decentralization was calculated using the center-command, and Marginal effects was calculated using the margins-command

Control variables included individual’s demographic, socioeconomic status, health behaviors and city-level climate variables in living areas. City dummy variables were also controlled

* P < 0.10; ** P < 0.05; *** P < 0.01

## Discussion

To the best of the authors’ knowledge, this is the first published article to elucidate the role of chronic disease in an association between air pollution and depressive symptoms within the Chinese population, who prefer to acknowledge poor mental conditions instead of mental illness. Using nationally representative data for the general Chinese middle- and old-aged population, this study found that exposure to air pollution was related to depression and that if an individual had a chronic disease, they were more vulnerable to the depressive symptoms effects of air pollution. These findings provide a comprehensive understanding of the extent that air pollution affects depression, which could provide valuable insights for the design of policies and promotional programs to enhance the quality of mental health and curb the negative effects of air pollution in China.

The results of a positive association between air pollution and depression are consistent with those of previous Chinese studies and results from Japan, Korea, Canada, and the Netherlands [8, 13–17], but are contradictory to results from Norway and Boston [27, 52]. This discrepancy could possibly be related to the intensity of air pollution [52, 53]. For example, air pollution in China occurs at some of the highest levels in the world: in Yale University’s 2016 Environmental Performance Index, China is ranked 109 out of 180 countries [54]. Therefore, insignificant results from countries with low pollution levels merely indicate that a minor amount of air pollution is not harmful with respect to depression, whereas levels are high in China, are causing health issues, and are continuing to rise.

This study also identifies susceptible subgroups and extends research on the subject of adverse mental health effects relating to air pollution by testing the assumption that individuals with chronic disease were more vulnerable to depressive symptoms effects relating to air pollution. It is of note that our study found that individuals with cardiovascular disease were more likely to report depressive symptoms related to air pollution compared to those without chronic disease, even when results were adjusted for other factors. While no studies have investigated the role of air pollution and depression relating to chronic disease, the relationship determined here is consistent with results of studies showing a relationship between (1) exposure to ambient air pollution and an increase in chronic mortality and morbidity [55–65]; (2) chronic disease and depressive symptoms [66].

Considering the large variation in optimal thresholds for identifying depressive symptoms in self-reported responses, this study adopted a continuous variable to measure depressive symptoms, instead of using a cut-off point to dichotomously distinguish depressive symptoms [67]. In doing so, caution is also warranted when attaching pathological labels to self-reported symptoms [68]. A dimensional scale with higher scores indicating more symptoms provides enhanced clarification of depressive symptoms [69], particularly for individuals with a Chinese cultural background where depressive symptoms are highly stigmatized [55, 70].

Nevertheless, the limitations of this study should be considered before discussing potential policy implications. The previous studies proved that other contaminants such as PM_10_ or PM_2.5_ may also be related to mental health effects [52]. However, use of these air pollution compounds was restricted in our analysis due to data constraints. A further limitation relating to data constraints is that only annual air pollution intensity data were available for use, which cannot be fully correlated with weekly depression symptoms. Although such data and associated results are useful in that depressive symptoms may result from accumulated exposure to air pollution [53], it is not possible to untangle short-term effects from long-term effects in this study. However, although caution should be taken when explaining the results, it is clear that high levels of SO_2_ and TSP within lead to depression.

An underlying endogeneity problem may exist if depressive symptoms also affect an individual’s chronic disease status, which would lead to a simultaneity issue [49, 71, 72]. For example, a depressed patient could become trapped in a negative cycle in which mental symptoms are exacerbated by the synergistic effect of stress and cardiovascular risk factors, or where a patient is vulnerable to acute cardiovascular events due to the synergistic effect of mental stress and an underlying atherothrombotic disorder [72]. It is considered that depressive symptoms can have an influence on the occurrence of hypertension, dyslipidemia, diabetes or high blood sugar, cardiovascular diseases, arthritis and asthma [71–83]. However, our individual level data relied on retrospective self-evaluation, which is potentially endogenous due to measurement errors. Although quasi-experimental or instrument variables could have corrected the bias [84] these corrections are now unavailable because of data limitations. The analyses presented here focused on a subsample of the population that excluded respondents suffering from chronic diseases stemming from depressive symptoms. To be specific, respondents with hypertension, dyslipidemia, diabetes or high blood sugar, cardiovascular diseases, arthritis and asthma were not included in subsample analysis [71–83]. As expected, our results show that compared to those without chronic disease, individuals with chronic disease not directly related to mental health were more likely to suffer from depression. Therefore, even when chronic disease is not considered to be a direct cause of depression in China, chronic disease influences an individual’s susceptibility to air pollution triggering depressive symptoms.

Given the above limitations, it is considered the results of this study can be used when establishing air pollution policies and policies related to both individual and public health concerns. In this respect, the adverse effects of air pollution on mental health should firstly be considered when establishing air pollution guidelines. If the unfavorable impacts of air pollution on mental health are overlooked, the national emission standard levels may be higher than optimal, which could result in excessive amounts of air pollutants being emitted. In addition, our findings provide a justification for establishing mental health interventions that target air pollution exposure. Public health policies should provide vulnerable people with information to help them cope with the adverse effects of air pollution. Furthermore, respondents with chronic disease found that depressive symptoms were particularly susceptible to fluctuations in air quality. Although all individuals are potentially exposed to ambient pollution, the evidence suggests that being of sound physical health cushions the depressive symptom effects of air pollution exposure. As such, basing policies on effects observed in the general population may be insufficient to protect vulnerable subgroups. The Chinese government needs to enhance and focus prevention strategies for those with chronic disease. For example, the Air Quality Health Index could provide different public advice for those with an elevated risk due to chronic disease when providing a summary of the air quality and advice to prevent adverse health effects [85].

## Conclusion

This study evaluated the association between air pollution and depressive symptoms and was based on nationally representative data relating to the Chinese middle- and old-aged population. The role of chronic disease with respect to air pollution and depressive symptoms was also estimated. Air pollution was found to be an important determinant of depressive symptoms, particularly for those with chronic disease. When an individual had a chronic disease, they were more vulnerable to the depressive symptoms effects of air pollution than those without chronic disease. Therefore, the adverse health effects of air pollution should be taken into consideration while establishing environmental and public health policies.

## Appendix

See Table 4.

**Table 4:** Association of air pollution emission per capita/per unit area and depressive symptoms and the role of chronic disease: Robustness test

Variables	Influence of SO_2_ emission intensity on depressive symptoms				Influence of TSP emission intensity on depressive symptoms			
	Model 1		Model 2		Model 3		Model 4	
	Marginal effects	Boot. Std.	Marginal effects	Boot. Std.	Marginal effects	Boot. Std.	Marginal effects	Boot. Std.
Association of air pollution emission per unit area (tons per square kilometer) and depressive symptoms and the role of chronic disease among respondents without mental-related chronic disease: Tobit model (N = 10,508)								
Log of SO_2_ emission per unit area	0.191	0.163	0.156	0.153	–	–	–	–
Log of TSP emission per unit area	–	–	–	–	0.490***	0.132	0.419***	0.161
Chronic disease	–	–	0.856***	0.107	–	–	0.861***	0.094
Log of SO_2_ emission per unit area × chronic disease	–	–	0.161*	0.093	–	–	–	–
Log of TSP emission per unit area × chronic disease	–	–	–	–	–	–	0.284**	0.124
Sigma(u)	2.088***	0.063	2.040***	0.079	2.092***	0.057	2.043***	0.072
Sigma(e)	3.453***	0.042	3.456***	0.040	3.449***	0.037	3.451***	0.040
Log likelihood	− 29,375		− 29,329		− 29,368		− 29,320	

	Model 5		Model 6		Model 7		Model 8	
	Marginal effects	Boot. Std.	Marginal effects	Boot. Std.	Marginal effects	Boot. Std.	Marginal effects	Boot. Std.
Association of air pollution emission per capita (tons per 10 thousand people) and depressive symptoms and the role of chronic disease among respondents without mental-related chronic disease: Tobit model (N = 10,508)								
Log of SO_2_ emission per capita	0.359***	0.122	0.332**	0.137	–	–	–	–
Log of TSP emission per capita	–	–	–	–	0.615***	0.121	0.564***	0.122
Chronic disease	–	–	0.860***	0.114	–	–	0.865***	0.088
Log of SO_2_ emission per capita × chronic disease	–	–	0.145	0.105	–	–	–	–
Log of TSP emission per capita × chronic disease	–	–	–	–	–	–	0.211*	0.118
Sigma(u)	2.089***	0.072	2.041***	0.057	2.093***	0.081	2.046***	0.076
Sigma(e)	3.452***	0.041	3.455***	0.034	3.447***	0.048	3.449***	0.037
Log likelihood	− 29,374		− 29,329		− 29,365		− 29,319	

Models 1–8 are estimated using the xi:xttobit-command in Stata 14. Decentralization was calculated using the center-command, and Marginal effects was calculated using the margins-command

Control variables included individual’s demographic, socioeconomic status, health behaviors and city-level climate variables in living areas. City dummy variables were also controlled

* P < 0.10; ** P < 0.05; *** P < 0.01

## Abbreviations

ANOVA: Analysis of Variance
CES-D: Center for Epidemiologic Studies Depression
CHARLS: China Health and Retirement Longitudinal Study
GDP: gross domestic product
NCMS: the rural new cooperative medical scheme
NO_2_: nitrogen dioxide
PM_10_: particle size smaller than 10 μm
PM_2.5_: particle size smaller than 2.5 μm
SO_2_: sulfur dioxide
TSP: total suspended particulates (particle size smaller than100 μm)
UEBMI: the urban employee-based basic medical insurance scheme
URBMI: the urban resident-based basic medical insurance scheme
WHO: World Health Organization
